# High prevalence of vaterite in sagittal otoliths causes hearing impairment in farmed fish

**DOI:** 10.1038/srep25249

**Published:** 2016-04-28

**Authors:** T. Reimer, T. Dempster, F. Warren-Myers, A. J. Jensen, S. E. Swearer

**Affiliations:** 1 Sustainable Aquaculture Laboratory – Temperate and Tropical (SALTT), School of BioSciences, University of Melbourne, Victoria 3010, Australia; 2 Research on the Ecology and Evolution of Fishes (REEF) laboratory, School of BioSciences, University of Melbourne, Victoria 3010, Australia; 3 Norwegian Institute for Nature Research (NINA), PO Box 5685 Sluppen, 7485 Trondheim, Norway

## Abstract

The rapid growth of aquaculture raises questions about the welfare status of mass-produced species. Sagittal otoliths are primary hearing structures in the inner ear of all teleost (bony) fishes and are normally composed of aragonite, though abnormal vaterite replacement is sometimes seen in the wild. We provide the first widespread evaluation of the prevalence of vaterite in otoliths, showing that farmed fish have levels of vaterite replacement over 10 times higher than wild fish, regardless of species. We confirm this observation with extensive sampling of wild and farmed Atlantic salmon in Norway, the world’s largest producer, and verify that vateritic otoliths are common in farmed salmon worldwide. Using a mechanistic model of otolith oscillation in response to sound, we demonstrate that average levels of vaterite replacement result in a 28–50% loss of otolith functionality across most of a salmonid’s known hearing range and throughout its life cycle. The underlying cause(s) of vaterite formation remain unknown, but the prevalence of hearing impairment in farmed fish has important implications for animal welfare, the survival of escapees and their effects on wild populations, and the efficacy of restocking programs based on captive-bred fish.

Aquaculture is the world’s fastest-growing food production industry, producing over 66 million tonnes of seafood per year^1^. Growth in production has been driven by increased use of intensive farming systems, creating health and welfare challenges, such as increased incidence of deformities, diseases and parasites. As such, welfare outcomes for farmed fish in aquaculture systems have received heightened attention. Guidelines in many jurisdictions are based on the ‘Five Freedoms’^2^, which specify freedom from discomfort, pain, injury, disease, fear and distress, and stipulate freedom to exhibit normal behaviours. Intensive culture systems are also widely used for re-stocking and conservation purposes^3^; if the performance of reared fish is compromised, the efficacy of such programs is likely diminished.

One approach for detecting potential welfare effects of animal culture systems is to document differences between wild and farmed populations. Recently, differences have been observed between the otoliths of farmed and wild fish^4^. Otoliths are calcium carbonate structures in the inner ear labyrinths of vertebrates. They are primitive and conserved sensory organs which contribute to hearing, balance, gravity sensation and linear acceleration, and are thus crucial for survival^5^. Otoliths are well studied in many wild fish species, as sagittal otoliths in particular provide an accurate record of age and growth. However, as the age and growth of farmed fishes is usually known, their otoliths are rarely studied. Sagittal otoliths are normally composed of aragonite, a polymorph of calcium carbonate, but otoliths with inclusions of vaterite, an alternate polymorph, also occur^6^. These ‘vaterite otoliths’ are transparent and larger than their aragonite counterparts (Fig. 1). Vaterite otoliths typically occur in fewer than 10% of wild fish, although there are exceptions^7^. Prevalence of vateritic otoliths in farmed fish may differ markedly from wild populations; several studies report vaterite in 50–60% of otoliths from hatchery-reared fish^8^,^9^,^10^,^11^,^12^,^13^. However, comparisons between the prevalence of vaterite otoliths in farmed and wild populations are few. No large-scale sampling has yet determined if vaterite is consistently more common in farmed populations, nor if the phenomenon is localised or widespread.

The causes of vaterite replacement in sagittal otoliths are unknown, but the consequences have been partially investigated. Oxman *et al.*^14^ used the auditory brainstem response (ABR) technique to test the hearing of Chinook salmon with vaterite otoliths against those with normal otoliths. Salmon with at least one vateritic otolith (having >33% of otolith planar area replaced by vaterite) experienced a loss in otolith functionality, especially at frequencies between 100–200 Hz. These results show important differences, but the authors used a coarse method of vaterite classification and measured hearing loss only in relation to sound pressure level despite evidence that salmonids rely on particle motion for sound detection^15^,^16^. It is therefore difficult to fully assess the effect of vaterite on otolith functionality despite their findings showing that no other part of the inner ear was affected by the presence of vaterite^14^. Consequently, the authors’ conclusions that having one or two vaterite otoliths impairs hearing to the same degree may be premature.

Modelling the hearing of fish may circumvent some limitations of experimental studies and provide a mechanistic understanding of how vaterite affects hearing. Lychakov and Rebane^17^ developed a model for predicting the effect of otolith mass-asymmetry on fish hearing. Based on the physics of otolith movement, it can be easily adapted to model sensitivity differences between vateritic and aragonitic otoliths. Models can also predict responses outside the frequency ranges of conventional hearing tests; for salmon, which can hear sounds as low as 0.1 Hz^18^, the effect of vaterite in the infrasound range is worth investigating. The model is based on particle motion rather than sound pressure, making it more suitable for assessing the effects of vaterite otoliths on salmonids^19^.

Here, we synthesise previous knowledge on vaterite otoliths, and provide a detailed and mechanistic understanding of their consequences. We analysed all known published comparisons of vaterite otoliths in wild and farmed populations to test if they are more prevalent in farmed fish. We conducted broad-scale sampling of farmed and wild Atlantic salmon throughout Norway, the world’s largest farmed salmon producer and a country with extensive wild populations, to eliminate confounding variables related to species, age and method of vaterite classification. To test if patterns were globally generalizable, we also sampled harvest-size farmed Atlantic salmon from Australia, Scotland, Canada and Chile. Finally, using a mechanistic model and data from Atlantic salmon of three different sizes, we examined how the extent of vaterite replacement affects hearing, including into the infrasound range, at different stages of the life history.

## Materials and Methods

### Analysis of existing literature for vaterite prevalence

We compiled previously published experimental data to assess whether farmed fish consistently have a higher prevalence of vaterite otoliths. Studies were found by searching for the keywords “vaterite”, “aberrant”, “abnormal” or “crystalline” in relation to the sagittal otoliths of any species using Web of Science and Google Scholar. To be included, papers must have (a) mentioned vaterite specifically or provided a sufficiently detailed description to allow its identification, and (b) provided data on vaterite otoliths from both farmed and wild populations of the same species in a similar area. In total, seven studies met the selection criteria. Where multiple age classes were available, only data from similar-aged fish was used. To control for different scoring methods, prevalence of vaterite otoliths was measured as the proportion of otolith samples containing any visible vaterite.

### Vateritic otoliths in farmed and wild Atlantic salmon

**Figure 2:**
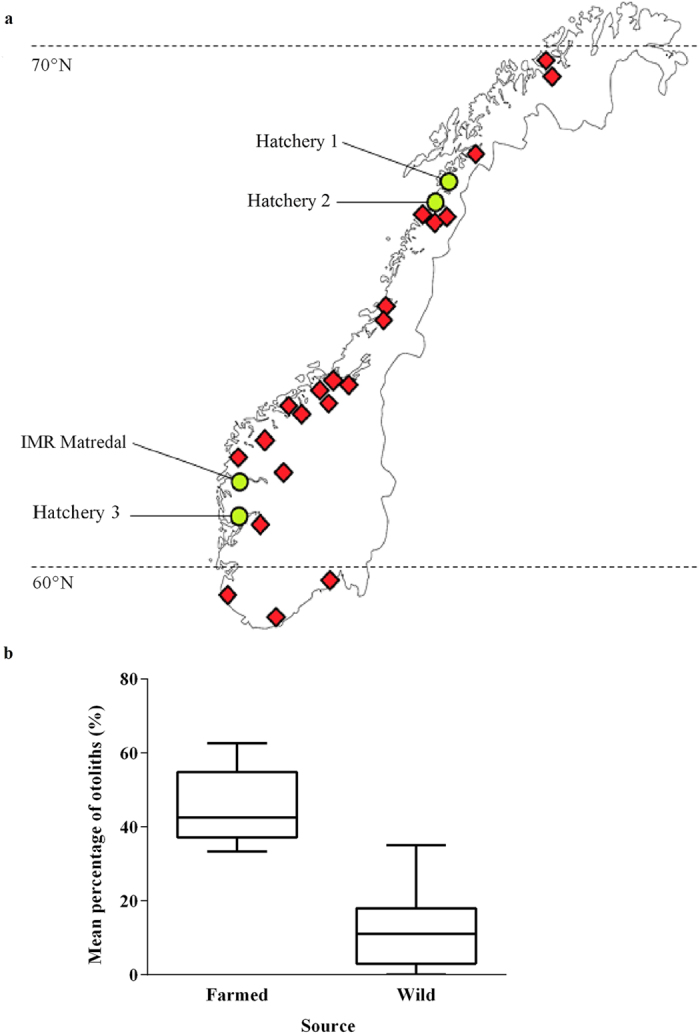
(**a**) Map of Norway showing sampling locations of farmed (green circle) and wild (red diamond) populations. (**b**) Prevalence of vaterite sagittal otoliths in farmed (n = 5) and wild (n = 23) Atlantic salmon populations. Map modified from https://pixabay.com/en/norway-map-country-europe-23574/.

Wild Norwegian Atlantic salmon were collected from 21 rivers across Norway between 1986 and 2010 (Fig. 2a). Otoliths from ten Atlantic salmon per river were removed, cleaned of adhering tissue with ethanol and dried. Fifty individuals from five populations of farmed Atlantic salmon were sourced from four Norwegian hatcheries in 2014. The salmon were frozen whole and sent to the Institute of Marine Research (IMR) in Matredal, Norway, where their otoliths were extracted, cleaned of adhering tissue and dried. Otoliths were photographed under a dissecting microscope at 10–30× magnification (depending on otolith size) and scored as ‘vaterite’ if any vaterite crystals were clearly identifiable or ‘aragonite’ if vaterite crystals were unclear or not present. Prevalence of vaterite was defined as the proportion of vaterite otoliths in the sample. Otoliths were measured for total area and vaterite area using ImageJ.

To measure the changes in vaterite prevalence and extent over time, we used otoliths from three cohorts of different sized fish: small (mean 33 g, range 17–53 g), medium (mean 334 g, range 108–582 g) and large (mean 4658 g, range 2775–6360 g), which correspond to approximate ages of 7, 12 and 18 months post-hatching, respectively. These salmon were reared at the Institute of Marine Research hatchery in Matre, Norway^20^,^21^,^22^. We also collected 10–60 harvest-sized fish (4–6 kg) from Australia, Scotland, Canada and Chile. Where possible, fish were purchased from multiple sources in each country to ensure samples came from several farms.

### Otolith oscillation and mass-asymmetry model

We used a model that was developed by Lychakov and Rebane^17^ to describe the differential oscillation of mass-asymmetrical otolith pairs in response to acoustic stimulation (1). This model was chosen for its explicit inclusion of otolith density, mass, planar area and volume, as these factors are changed by vaterite replacement.


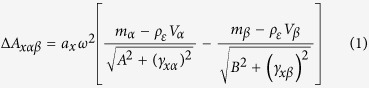


where *A_x_* is oscillation amplitude, α and β are aragonite and vaterite otoliths respectively (replacing the original R and L notation), *a_x_* is the oscillation of water and the fish’s body, ω is the wavelength of sound, *m* is mass in mg, 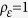 g/cm^3^ is endolymph density, *V* is otolith volume, 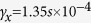 is a friction coefficient, 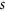 is otolith area in mm^2^, and


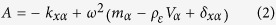



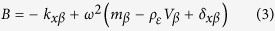


where 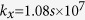 is the stiffness coefficient for a saccular otolith and 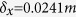 is the additional mass of an ellipsoid otolith. Otolith volumes were calculated by:


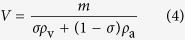


where 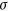 is the proportion of planar area replaced with vaterite (expressed as a decimal), ρ_a_ = 2.95 g/cm^3^ is the density of bioaragonite and ρ_v_ = 2.54 g/cm^3^ is the density of biovaterite^23^.

To realistically model otolith oscillation for farmed fish, model parameters were estimated using the otoliths of three different sizes of fish (Table 1). Samples were weighed (±0.05 mg for medium and large otoliths, ±0.0005 mg for small otoliths) and *m*_α_ was determined experimentally as the average mass of all aragonite otoliths for a given fish weight. *m*_β_ was determined by:


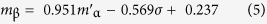


where *m*′_α_ is the mass of the corresponding aragonite otolith. Similarly, aragonite planar area was determined as the average area of aragonite otoliths for each size of fish. The planar area of vaterite otoliths was determined by:


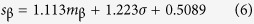


Only one fully aragonite otolith was found among the large fish, so Eqs 5 and 6 were used to estimate the corresponding aragonite otolith parameters from vaterite samples.

This work was conducted in accordance with the laws and regulations of the Norwegian Regulation on Animal Experimentation 1996. Experimental protocols were approved by the Norwegian Animal Research Authority.

### Data analysis

#### Analysis of previous literature for vaterite prevalence

We calculated effect size by dividing the proportion of otolith samples affected by vaterite in populations of farmed fish by those of wild fish. Statistical significance was tested using a paired t-test with studies as replicates.

#### Vateritic otoliths in farmed and wild Atlantic salmon

Prevalence of vaterite was analysed with an unpaired t-test with the proportion of otoliths affected by vaterite in each area as replicates.

The proportion of vateritic otoliths in a sample was used to represent the probability of the onset of vaterite formation. This probability was assumed to be independent between left and right labyrinths. To determine if vaterite formation is biased towards one side, proportions of vateritic left and right otoliths were analysed with a paired t-test across all sample sets. The expected number of fish with two vateritic otoliths was determined using the product of the proportions of vateritic left and right otoliths, and was analysed against observed numbers with a Chi-square test.

## Results

### Analysis of previous literature for vaterite prevalence

Existing studies indicated that farmed populations had 10.4 times higher incidence of vaterite sagittal otoliths (Table 2, p < 0.001). Levels of vaterite did not vary consistently with year of study or species. Overall, 8.6% of wild otoliths and 48.7% of farmed otoliths had some level of vaterite replacement.

### Vateritic otoliths in farmed and wild salmon

Norwegian Atlantic salmon yearlings raised in hatcheries had 3.7 times higher incidence of vateritic otoliths than wild populations (Fig. 2b, p < 0.0001). The percentage of Norwegian fish affected increased with fish size, with 66% of small fish, 75% of medium fish and 100% of large fish having at least one vateritic otolith. Average level of vaterite replacement also increased with fish size, from 47% in small fish to 56% in medium fish and 88% in large fish, so that large fish had 1.9 times the vaterite replacement of small fish (Table 1). Left otoliths were more likely to be vateritic than right otoliths, with 58% and 52% of otoliths showing vaterite formation, respectively (df = 64, p = 0.005). Of all fish sampled, 42% had two vateritic otoliths, which was higher than the expected proportion of 39% (df = 1, p = 0.004). Incidence of vateritic otoliths was similarly high in populations of harvest-size farmed Atlantic salmon from Australia (57% of otoliths vateritic, n = 102), Scotland (58%, n = 38), Canada (30%, n = 64) and Chile (64%, n = 28), as well as rainbow trout from Chile (48%, n = 17).

### Otolith oscillation and mass-asymmetry model

**Figure 3:**
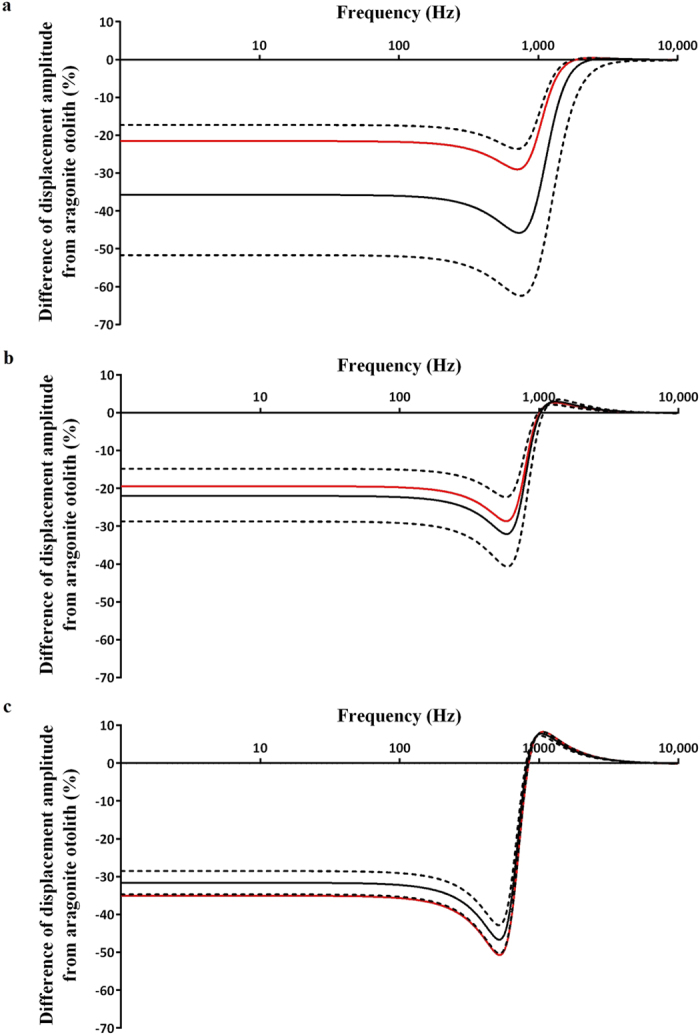
Loss of otolith oscillation amplitude due to vaterite replacement at varying sound frequencies for (**a**) small, (**b**) medium and (**c**) large salmon. Black lines represent average levels of vaterite replacement across all fish at 64 ± 22% planar area [Mean ± SD]. Red lines represent the effect of mean levels of vaterite replacement for small (47%), medium (56%), and large fish (88%).

Vaterite otoliths were on average 17% larger and 8% lighter than their aragonite counterparts (Table 1). All otoliths with vaterite replacement lost oscillation amplitude compared to their aragonite counterparts, and increasing severity of vaterite replacement consistently resulted in a larger loss of amplitude (Fig. 3). When the level of vaterite replacement was the same across otolith sizes, small otoliths lost the most oscillation amplitude, with a maximum of 46% lost at mean replacement (Fig. 3, black lines). Small otoliths were also greatly influenced by changes in vaterite replacement, such that their maximum level of oscillation loss varied by 39% within one standard deviation from the mean. Large otoliths were least affected by changes in vaterite replacement, with oscillation loss varying by only 7.5% within the same interval. As observed levels of vaterite replacement increased with otolith size (Table 1), maximum average oscillation loss was 51% at 522 Hz for large fish, 29% at 583 Hz for medium fish, and 29% at 708 Hz for small fish (Fig. 3, red lines). In the infrasound range (1–20 Hz) small, medium and large otoliths with overall mean vaterite replacement lost 22%, 19% and 35% of oscillation amplitude, respectively.

## Discussion

Fish raised in hatcheries are up to 10 times more likely to have vateritic sagittal otoliths than their wild counterparts, and may experience hearing loss as a result. Previous research has found differences in the prevalence of vaterite otoliths of 8.4–55% between wild and farmed fish (Table 2). Our results show a difference of 33%; farmed Norwegian Atlantic salmon have levels of vaterite 3.7 times higher than their wild equivalents. This result is not limited to Norway, as farmed Atlantic salmon from Australia, Scotland, Canada and Chile also have high levels of vaterite replacement. Vaterite otoliths have decreased oscillation amplitude in response to sound, which impairs functionality relative to aragonite otoliths. When vaterite replacement is consistent across all sizes, the effect is more pronounced in smaller otoliths. However, larger otoliths have higher average vaterite coverage, so large fish would suffer the most impairment. This loss of oscillation amplitude due to vaterite occurs across the majority of the salmon hearing range, including the infrasound.

Cultured fish worldwide may lose hearing sensitivity due to the farming process. The primary hearing range of salmon is between 100 and 300 Hz, with a maximum tested frequency of 1000 Hz and a minimum of 0.1 Hz^14^,^18^. Our results show that the more extensive an otolith’s vaterite coverage, the more its function is likely to be impaired. This implies that fish with two vaterite otoliths will be more affected than those with only one, but this is not supported by experimental evidence^14^. This difference may be due to technical limitations inherent in the non-invasive ABR technique and the coarse method of vaterite classification used by Oxman *et al.*^14^. Testing the sensitivity of individual ears using our more precise measurement of vaterite coverage may discover the cause of the discrepancy. Difficulties could also stem from the measurement of sound pressure rather than particle motion, which is the more relevant variable and can produce vastly different results^24^. Finally, the difference in results could be indicative of a compensatory mechanism in salmon affected by vaterite. However, this mechanism evidently fails to fully compensate for hearing loss due to vaterite replacement and may only be present in extreme cases. Further study of a compensatory mechanism would supplement our limited understanding of salmonid hearing and how it is affected by culture systems.

Species with large otoliths are likely to be more severely affected by changes to the size, shape and density of their otoliths due to vaterite replacement (Fig. 3C). Some species also have an “indirect” pathway for sound detection, where acoustic pressure is detected by the swim bladder, but this signal is still mediated by the otoliths^16^,^25^. Therefore, even species with secondary hearing mechanisms may be susceptible to hearing impairment from vaterite replacement. Hearing loss may be especially relevant in the infrasound range, as many underwater mechanical sounds (such as those produced by swimming predators or struggling prey) are below 20 Hz^15^. The results from our model show that vaterite may impair sensitivity in the infrasound as much as in the rest of the salmonid hearing range, but hearing impairment has never been tested below 100 Hz. With further testing, vaterite replacement could explain why hatchery salmon demonstrate decreased predator evasion and increased mortality compared to wild fishes^3^,^26^.

Vaterite formation may also impair hearing directionality by creating mass asymmetry or, if only one otolith is required for directional hearing, changing the way the otolith moves in relation to its associated hair cells^27^,^28^,^29^. Our results show that vaterite formation results in a larger, lighter otolith and may be slightly biased towards the left ear, but also suggest that once one otolith has begun vaterite formation, the other otolith is more likely to as well. This implies that a large mass asymmetry is rare even in fish with vateritic otoliths. Lychakov and Rebane^17^ estimate that serious problems in directionality may only occur where mass asymmetry between otoliths is >0.2, and only one individual (<0.1%) in this study achieved this. Vaterite replacement also changes the density, brittleness, size, and shape of the otolith; density in particular may have a strong effect on directionality^30^. However, the lagena and utricle also contribute to sound localisation, and affected fish may be able to develop compensatory mechanisms. Further study is required to determine the individual and synergistic effects of vaterite’s properties on the full range of otolith functions.

The high incidence of vaterite otoliths and their effect on hearing contravenes two of the ‘Five Freedoms’, thereby lowering the welfare of farmed fishes^2^,^31^. Although our results cannot directly quantify the nature or extent of hearing loss, we provide a mechanistic understanding and basis from which to study its potential behavioural impacts. Impairment of a fish’s hearing due to vaterite replacement may prevent the expression of normal behaviour, which is especially relevant in farmed species that communicate using sound (e.g. Yellow and Japanese croaker^32^; Nile tilapia^33^). As deformity is a consequence of disease, the formation of vateritic sagittal otoliths infringes on the freedom from pain, injury or disease. Other physical deformities are common in farmed salmon, particularly in the jaw and spine^34^, and research into methods of prevention and treatment is ongoing^35^. As vaterite appears to be permanent once formation has begun, future control efforts should focus on prevention. Further research is needed to investigate the cause(s) of vaterite formation in the otoliths of farmed fish.

Loss of hearing in captive-bred fishes could have negative ecological impacts worldwide. Many wild rivers are deliberately stocked with hatchery-reared salmon: in 2013, 5 × 10^9^ juveniles were released into the Northern Pacific Ocean alone^36^, and in some areas reared juveniles comprise over 70% of returning salmon^11^. However, ocean survival rate of reared salmon is low, varying between 1% and 15%^37^,^38^. Vaterite replacement may contribute to this low return rate by impairing navigation and habitat selection important for survival^27^. Lack of hearing sensitivity in the infrasound range may also reduce the effectiveness of acoustic dams, which rely on salmon showing a strong aversion to infrasound^15^. Vaterite has never been investigated in salmon returning from the ocean, or with respect to predator aversion and mortality. Future research into these areas could shed light on whether hearing impairment is one of the underlying causes of differential survival and reproductive success between hatchery-produced and wild fish.

### Data accessibility

The dataset supporting this article have been uploaded as part of the supplementary material.

## Additional Information

**How to cite this article**: Reimer, T. *et al.* High prevalence of vaterite in sagittal otoliths causes hearing impairment in farmed fish. *Sci. Rep.* **6**, 25249; doi: 10.1038/srep25249 (2016).

## Supplementary Material

Supplementary Dataset 1

## Figures and Tables

**Figure 1:**
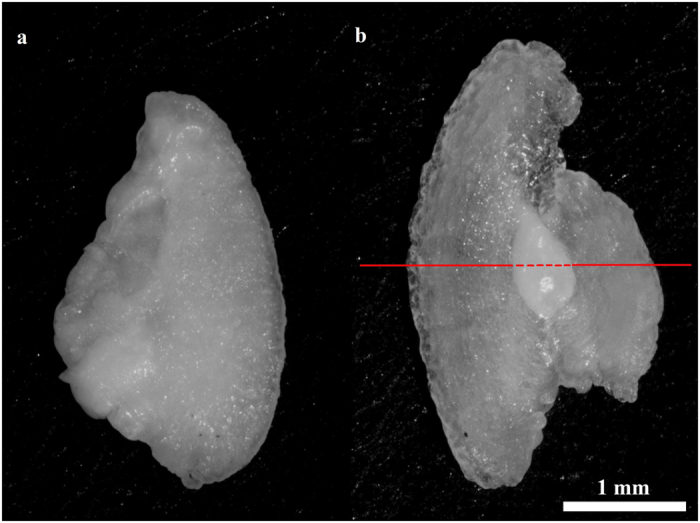
Sagittal otoliths from a farmed Atlantic salmon juvenile. The left otolith (**a**) is entirely aragonite. The right otolith (**b**) is approximately 90% vaterite by planar area, and the red line marks the border between the aragonite core (dashed) and the surrounding vaterite (solid).

**Table 1:** Summary of model parameters calculated for aragonite and vaterite otoliths using three different sizes of fish.

Parameter	Fish size		
	Small	Medium	Large
Aragonite otolith mass (*m*_α_, mg)	0.83	3.2	9.2
Vaterite otolith mass (*m*_β_, mg)	0.76	3.0	8.4
Aragonite otolith planar area (*s*_α_, mm^2^)	1.7	4.1	8.4
Vaterite otolith planar area (*s*_β_, mm^2^)	1.9	4.5	11
Mean vaterite replacement (σ, %)	47	56	88

**Table 2:** Analysis of previous literature comparing vaterite prevalence in sagittal otoliths of farmed and wild populations.

Study (data set)	Species	Vaterite Prevalence (%)			
		Wild	Farmed	Difference	Effect size
Watson^8^	*Clupea harengus*	6.1	60	53.9	9.8
Peck^9^	*Oncorhynchus kisutch*	1.4	55.9	54.5	39.9
Bowen II *et al.*^10^ (5–12 yr)	*Salvelinus namaycush*	24	48	24	2
Sweeting *et al.*^11^ (1997)	*Oncorhynchus kisutch*	7	52	45	7.4
Sweeting *et al.*^11^ (1998)	*Oncorhynchus kisutch*	8.4	56.6	48.2	6.7
Tomás & Geffen^23^	*Clupea harengus*	5.5	13.9	8.4	2.5
Sweeting *et al.*^12^	*Oncorhynchus kisutch*	11.8	52.9	41.1	4.5
Brown *et al.*^13^	*Oncorhynchus mykiss*	5	50	45	10

Sources^11^,^12^,^13^,^23^

## References

[b1] FAO. *The State of World Fisheries and Aquaculture: Opportunities and Challenges*. (Food and Agriculture Organisation of the United Nations, Fisheries and Aquaculture Department, 2014).

[b2] Farm Animal Welfare Council. *Report on Farm Animal Welfare in Great Britain: Past, Present and Future*. (2009). Accessed August, 2015. Available at https://www.gov.uk/government/publications/fawc-report-on-farm-animal-welfare-in-great-britain-past-present-and-future.

[b3] Einum S. & Fleming I. A. Implications of stocking: ecological interactions between wild and released salmonids. Nord. J. Freshw. Res. 75, 56–70 (2001).

[b4] Vinagre C., Maia A., Amara R. & Cabral H. N. Anomalous otoliths in juveniles of common sole, *Solea solea*, and Senegal sole, *Solea senegalensis*. Mar. Biol. Res. 10, 523–529 (2014).

[b5] Popper A. N. & Lu Z. Structure-function relationships in fish otolith organs. Fish. Res. 46, 15–25 (2000).

[b6] Carlström D. A crystallographic study of vertebrate otoliths. Biol. Bull. 125, 441–463 (1963).

[b7] Morat F. *et al.* What can otolith examination tell us about the level of perturbations of Salmonid fish from the Kerguelen Islands? Ecol. Freshw. Fish 17, 617–627 (2008).

[b8] Watson J. E. Determining the age of young herring from their otoliths. Trans. Am. Fish. Soc. 93, 11–20 (1964).

[b9] Peck T. H. Differentiation of hatchery and stream juvenile coho salmon (*Oncorhynchus kisutch*) from Washington and Oregon by the use of scales and otoliths. (University of Washington., 1970).

[b10] Bowen C. A. II, Bronte C. R., Argyle R. L., Adams J. V. & Johnson J. E. Vateritic sagitta in wild and stocked lake trout: Applicability to stock origin. Trans. Am. Fish. Soc. 128, 929–938 (1999).

[b11] Sweeting R. M., Beamish R. J., Noakes D. J. & Neville C. M. Replacement of Wild Coho Salmon by Hatchery-Reared Coho Salmon in the Strait of Georgia over the past Three Decades. North Am. J. Fish. Manag. 23, 492–502 (2003).

[b12] Sweeting R. M., Beamish R. J. & Neville C. M. Crystalline otoliths in teleosts: Comparisons between hatchery and wild coho salmon (*Oncorhynchus kisutch*) in the Strait of Georgia. Rev. Fish Biol. Fish. 14, 361–369 (2004).

[b13] Brown A. D., Sisneros J. A., Jurasin T., Nguyen C. & Coffin A. B. Differences in lateral line morphology between hatchery- and wild-origin steelhead. PLoS One 8, e59162 (2013). 23554988 10.1371/journal.pone.0059162 PMC3598794

[b14] Oxman D. S. *et al.* The effect of vaterite deposition on sound reception, otolith morphology, and inner ear sensory epithelia in hatchery-reared Chinook salmon (*Oncorhynchus tshawytscha*). Can. J. Fish. Aquat. Sci. 64, 1469–1478 (2007).

[b15] Sand O. *et al.* Detection of infrasound in fish and behavioral responses to intense infrasound in juvenile salmonids and European silver eels: a minireview. Paper presented at American Fisheries Society Symposium: Behavioral Technologies for Fish Guidance (ed. Coutant C. C.) 183 (American Fisheries Society, 2001).

[b16] Webb J. F., Popper A. N. & Fay R. R. Fish Bioacoustics. Springer Handbook of Auditory Research. Springer Science, NY, USA (2008)

[b17] Lychakov D. V. & Rebane Y. T. Fish otolith mass asymmetry: morphometry and influence on acoustic functionality. Hear. Res. 201, 55–69 (2005). 15721561 10.1016/j.heares.2004.08.017

[b18] Sand O. & Karlsen H. E. Detection of infrasound and linear acceleration in fishes. Philos. Trans. R. Soc. B Biol. Sci. 355, 1295–1298 (2000). 10.1098/rstb.2000.0687 PMC1692823 11079418

[b19] Hawkins A. D. & Johnstone A. D. F. The hearing of the Atlantic salmon, Salmo salar. J. Fish Biol. 13, 655–673 (1978).

[b20] Fjelldal P. G., Hansen T. & Albrektsen S. Inadequate phosphorus nutrition in juvenile Atlantic salmon has a negative effect on long-term bone health. Aquaculture 334, 117–123 (2012).

[b21] Samsing F., Solstorm D., Oppedal F., Solstorm F. & Dempster T. Gone with the flow: current velocities mediate parasitic infestation of an aquatic host. Int. J. Parasitol. 45, 559–565 (2015). 25917926 10.1016/j.ijpara.2015.03.006

[b22] Warren-Myers F., Dempster T., Fjelldal P. G. & Hansen T. & Swearer, S. E. Immersion during egg swelling results in rapid uptake of stable isotope markers in salmonid otoliths. Can. J. Fish. Aquat. Sci. 72, 722–727 (2015).

[b23] Tomás J. & Geffen A. J. Morphometry and composition of aragonite and vaterite otoliths of deformed laboratory reared juvenile herring from two populations. J. Fish Biol. 63, 1383–1401 (2003).

[b24] Radford C. A., Montgomery J. C., Caiger P. & Higgs D. M. Pressure and particle motion detection thresholds in fish: a re-examination of salient auditory cues in teleosts. J. Exp. Biol. 215, 3429–3435 (2012). 22693030 10.1242/jeb.073320

[b25] Rogers P. H., Popper a. N., Hastings M. C. & Saidel W. M. Processing of acoustic signals in the auditory system of bony fish. J. Acoust. Soc. Am. 83, 338–349 (1988). 3343448 10.1121/1.396444

[b26] Jonsson N., Jonsson B. & Hanson L. P. The marine survival and growth of wild and hatchery-reared atlantic salmon. J. Appl. Ecol. 40, 900–911 (2003).

[b27] Gagliano M., Depczynski M., Simpson S. D. & Moore J. A. Dispersal without errors: symmetrical ears tune into the right frequency for survival. Proc. R. Soc. B Biol. Sci. 275, 527–534 (2008). 10.1098/rspb.2007.1388 PMC2596807 18077258

[b28] Bignami S., Enochs I. C., Manzello D. P., Sponaugle S. & Cowen R. K. Ocean acidification alters the otoliths of a pantropical fish species with implications for sensory function. Proc. Natl. Acad. Sci. 110, 7366–7370 (2013). 23589887 10.1073/pnas.1301365110 PMC3645591

[b29] Rogers P. H. & Zeddies D. G. In Fish bioacoustics 233–252 (Springer, 2008).

[b30] Kondrachuk A. V. Mass and mechanical sensitivity of otoliths. Adv. Sp. Res. 32, 1521–1526 (2003). 10.1016/S0273-1177(03)90390-5 15000122

[b31] Damsgård B., Juell J.-E. & Braastad B. O. Welfare in farmed fish. (Fiskeriforskning (Norwegian Institute of Fisheries and Aquaculture Research), 2006).

[b32] Connaughton M. A., Lunn M. L., Fine M. L. & Tayor M. H. Characterization of sounds and their use in two sciaenid species: weakfish and Atlantic croaker. in Listening to Fish: Passive Acoustic Applications in Marine Fisheries 15–19 (2002).

[b33] Longrie N. *et al.* Behaviours Associated with Acoustic Communication in Nile Tilapia (*Oreochromis niloticus*). PLos One 8, 13 (2013). 10.1371/journal.pone.0061467 PMC3631236 23620756

[b34] Berg A., Yurtseva A., Hansen T., Lajus D. & Fjelldal P. G. Vaccinated farmed Atlantic salmon are susceptible to spinal and skull deformities. J. Appl. Ichthyol. 28, 446–452 (2012).

[b35] Fjelldal P. G. *et al.* Vertebral deformities in farmed Atlantic salmon (*Salmo salar* L.) - etiology and pathology. J. Appl. Ichthyol. 28, 433–440 (2012).

[b36] North Pacific Anadromous Fish Commission (NPAFC). NPAFC Pacific salmonid hatchery release statistics (updated 19 December 2014). (2014). North Pacific Anadromous Fish Commission: Vancouver. Accessed August, 2015. Available at www.npafc.org/new/science_statistics.html.

[b37] Beamish R. J. *et al.* Wild chinook salmon survive better than hatchery salmon in a period of poor production. Environ. Biol. Fishes 94, 135–148 (2012).

[b38] Moore M., Berejikian B. A. & Tezak E. P. Variation in the early marine survival and behavior of natural and hatchery-reared Hood Canal steelhead. PLoS One 7, e49645 (2012). 23185393 10.1371/journal.pone.0049645 PMC3501469

